# BioSamples database: an updated sample metadata hub

**DOI:** 10.1093/nar/gky1061

**Published:** 2018-11-08

**Authors:** Mélanie Courtot, Luca Cherubin, Adam Faulconbridge, Daniel Vaughan, Matthew Green, David Richardson, Peter Harrison, Patricia L Whetzel, Helen Parkinson, Tony Burdett

**Affiliations:** EMBL-EBI, Wellcome Genome Campus, Hinxton CB10 1SD, UK

## Abstract

The BioSamples database at EMBL-EBI provides a central hub for sample metadata storage and linkage to other EMBL-EBI resources. BioSamples has recently undergone major changes, both in terms of data content and supporting infrastructure. The data content has more than doubled from around 2 million samples in 2014 to just over 5 million samples in 2018. Fast, reciprocal data exchange was fully established between sister Biosample databases and other INSDC partners, enabling a worldwide common representation and centralization of sample metadata. The BioSamples platform has been upgraded to accommodate anticipated increases in the number of submissions via GA4GH driver projects such as the Human Cell Atlas and the EGA, as well as from mirroring of NCBI dbGaP data. The BioSamples database is now the authoritative repository for all INSDC sample metadata, an ELIXIR Deposition Database for Biomolecular Data and the EMBL-EBI sample metadata hub. To support faster turnaround for sample submission, and to increase scalability and resilience, we have upgraded the BioSamples database backend storage, APIs and user interface. Finally, the website has been redesigned to allow search and retrieval of records based on specific filters, such as ‘disease’ or ‘organism’. These changes are targeted at answering current use cases as well as providing functionalities for future emerging and anticipated developments. Availability: The BioSamples database is freely available at http://www.ebi.ac.uk/biosamples. Content is distributed under the EMBL-EBI Terms of Use available at https://www.ebi.ac.uk/about/terms-of-use.

## INTRODUCTION

**Figure 1.**
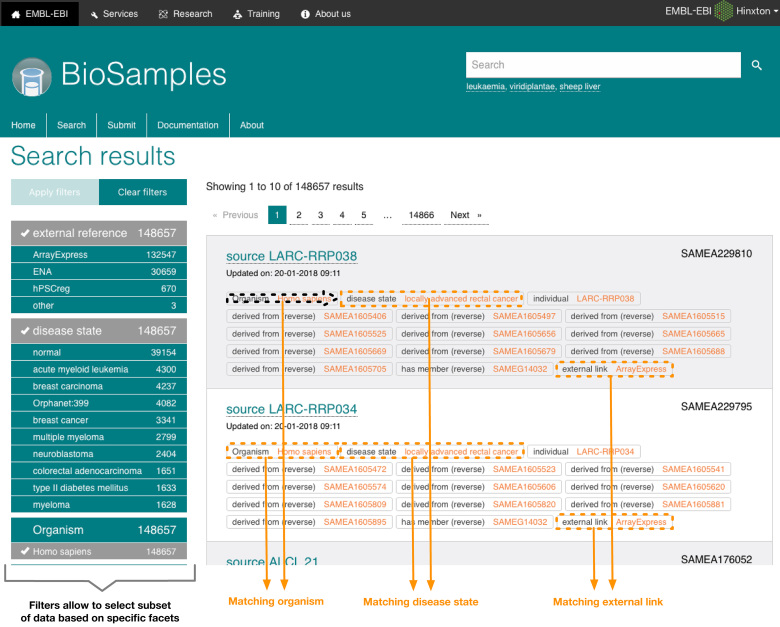
A search query for ‘Homo sapiens’ samples with ‘disease state’ associated metadata and links to external database visualized in the new BioSamples user interface. https://www.ebi.ac.uk/biosamples/samples?filter=attr:Organism:Homo+sapiens&filter=attr:disease+state&filter=attr:external+reference.

Several data archives store assay results on biological samples at EMBL-EBI. These include, amongst others, ArrayExpress (1) for functional genomics data, the European Nucleotide Archive (ENA) (2) for nucleotide sequencing information or the European Genome-phenome Archive (EGA) (3) for personally identifiable genotypic and phenotypic data. The BioSamples database provides a centralized way to describe information about the biological samples that undergo those experiments, such as for example organism, material sampled, disease states and relationship between samples. This allows for consistent representation of samples metadata, in a separate dedicated resource, which can then be linked to and from to track biosamples across other repositories and facilitate interpretation of data resulting from experiments. Using the BioSamples database users need only enter samples information once, minimizing burden on submitter and maximizing consistency across archives. Since our last publication in 2014 (4), the BioSamples database has undergone major changes. The data content has more than doubled from around 2 million samples in 2014 to just over 5 million samples in 2018. Fast, reciprocal data exchanges were fully established between sister Biosample databases at the other International Nucleotide Sequence Database Collaboration (INSDC) partners, the National Center for Biotechnology Information (NCBI) (5) and the DNA Data Bank of Japan (DDBJ) (6), enabling a worldwide common representation of sample metadata. Consequently the BioSamples database has become the authoritative repository for all INSDC sample metadata. It plays a vital role in data coordination, acting as the sample metadata repository for many biomedical projects including the Functional Annotation of Animal Genomes (FAANG) (7), the European Bank for induced pluripotent Stem Cells (EBiSC) (8) and the Human Induced Pluripotent Stem Cell Initiative (HipSci) (9,10). Data growth has also been driven by the ever more important role BioSamples is playing as an ELIXIR Deposition Database for Biomolecular Data (11) and as the EMBL-EBI hub for sample metadata. The BioSamples database is now the destination for samples from ELIXIR-EXCELERATE use case projects ranging from plant phenotyping to marine metagenomics, https://www.elixir-europe.org/about-us/how-funded/eu-projects/excelerate, as well as several other community efforts including the Global Alliance for Genomics and Health (GA4GH) (12) and the ELIXIR Bioschemas project, http://bioschemas.org, which can now be accessed via the Google dataset search tool, https://toolbox.google.com/datasetsearch/search?query=biosamples. Finally, the BioSamples platform has been upgraded to accommodate anticipated increases in the number of submissions via GA4GH driver projects such as Human Cell Atlas (HCA) (13) and the EGA, as well as from mirroring of NCBI database of Genotypes and Phenotypes (dbGaP) (14) data. The website has been redesigned, supporting the combination of multiple filters, to provide users a much improved search experience as shown on Figure 1, as well as allow retrieval based on specific filters, such as ‘disease’ or ‘organism’.

## A SAMPLE METADATA HUB

To streamline the process of submitting and updating data, the Unified Submission Interface (USI) solution has been developed to provide a single entry point to all EMBL-EBI archives. As part of the USI deployment, the metadata of samples submitted to EMBL-EBI is brokered to the BioSamples database. The corresponding BioSamples ID created during submission is returned to USI and is available for use in further data submissions to EMBL-EBI archives. For example, samples registered with the BioSamples database can be referenced in a submission to the ENA and sequence read data attached to this sample. Using BioSamples accessions as unique identifiers has several advantages. Firstly, all information can be integrated and traced back using this unique ID; secondly the process of submitting data to EMBL-EBI archives is vastly simplified, especially in multi-omic studies, as there is a single submission step for sample metadata; and finally, metadata is easier to maintain and remains consistent even when samples are used between different archives. To enable this simpler submission process and to support single sign-on to EMBL-EBI resources, the BioSamples database is now using a new Authentication, Authorization and Profile (AAP), https://aai.ebi.ac.uk, system. This provides secure sample submission and retrieval, and allows login using an account from a wide variety of identity providers, including OpenID, https://openid.net/ (e.g. login with a Google account), educational and institutional accounts that are part of the edugain federation, https://technical.edugain.org and ORCID, https://orcid.org. The AAP provides a centralised role management function, which allows the management of permissions to be coordinated in a single place across many ELIXIR services. Since January 2018, every sample in the BioSamples database is associated with an AAP domain (a user or group of users with shared permissions). Users with read or write access to that domain can see or update the sample’s original metadata, even in cases where the sample is private (for example, because it is being held until after the release of a corresponding publication). Editing of samples can be restricted to specific users, or permissions can be shared within groups for specific projects. For example, FAANG requires all the curators in its dedicated Data Coordination Centre to be able to update all FAANG samples, independently of who the original submitter was (15). AAP domains are used not only for sample submission but also in BioSamples curation, using a new framework we describe below which opens up the possibility of tracking curation events and filtering only those of interest to the user, a feature requested by projects and model organism databases such as VectorBase (16).

**Figure 2.**
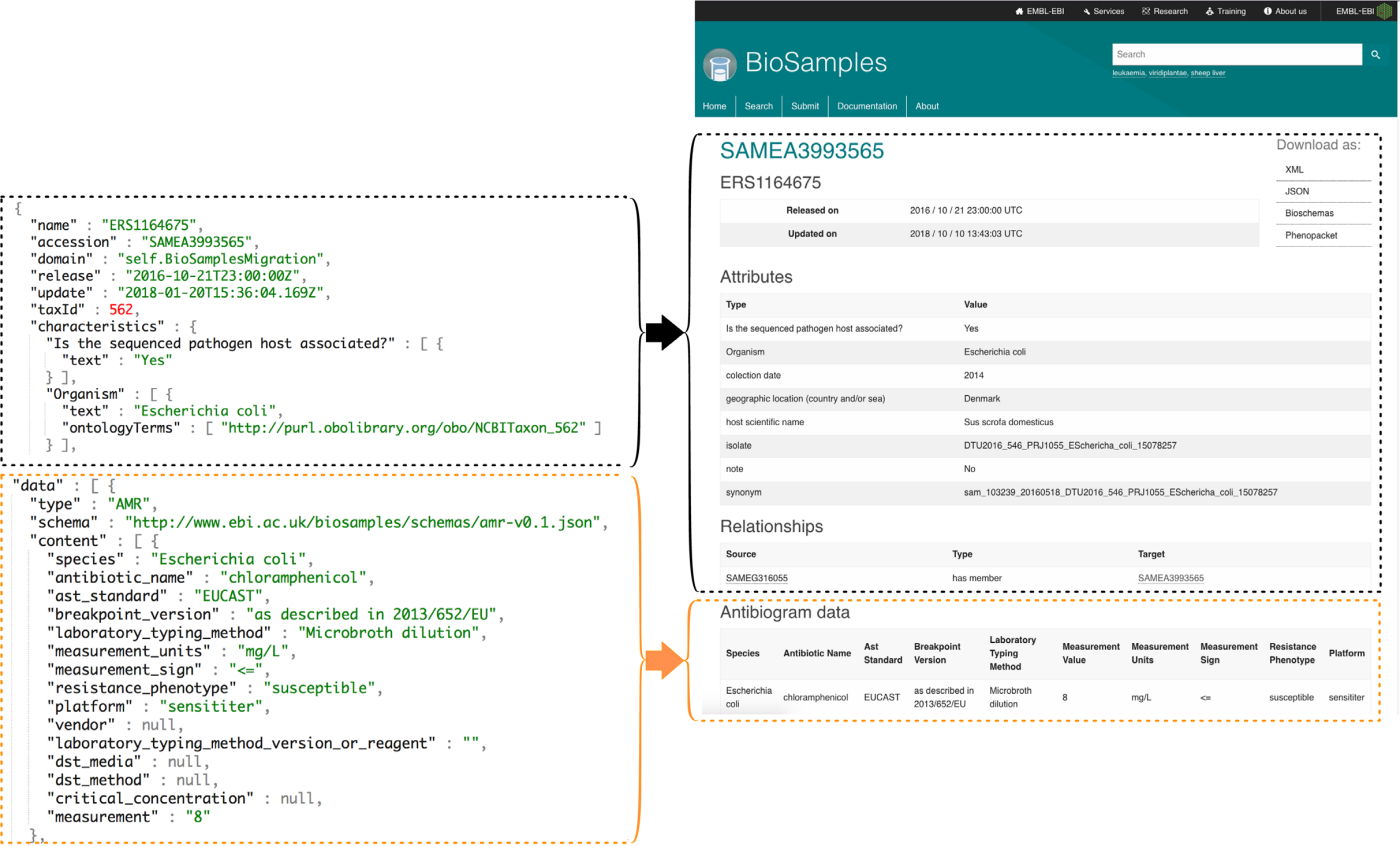
An example of user-supplied JSON block representing AMR data. On the left hand side the JSON file and on the right side the rendering in the BioSamples UI. The block boxes contain the attribute name/value pairs, the orange boxes frame the AMR data Some attributes omitted from for clarity.

Since 2014, we have seen that the diversity of user requests to the BioSamples database has increased concomitantly with data submissions. Automation around sample data collection as well as growth of high throughput phenotyping methods mean sample metadata is available for more samples and is richer and more complex. We currently have requests to support additional metadata for plant phenotyping, as well as description of non-primary cell lines according to the Cellosaurus (17) template for organoid cell lines submitted by the Wellcome Trust Sanger Institute. In addition to those, the COMPARE pilot project, http://www.compare-europe.eu, requires a large enough antibiogram dataset to develop new machine learning algorithms to determine antimicrobial resistance (AMR). AMR data is typically captured in a large table summarizing assay results evaluating antimicrobial resistance. In order to represent this data for downstream interpretation, we are now including additional structure in sample records. We have extended the BioSamples data model to optionally allow user-supplied blocks of JSON to be added to samples, alongside the existing attribute name/value pairs, as shown on Figure 2.

To ensure that this metadata is accessible to other users, we require that the supplied content be attached to a sample using the *data* attribute name with a specific *type* value, and that the content validates against a JSON schema corresponding to that type. The BioSamples database is now the unique centralized repository where AMR metadata can be accessed for further analysis, and the mechanism chosen for data representation will support the addition of other types, such as cell line information or plant phenotyping data.

## EVOLVING USER NEEDS

**Figure 3.**
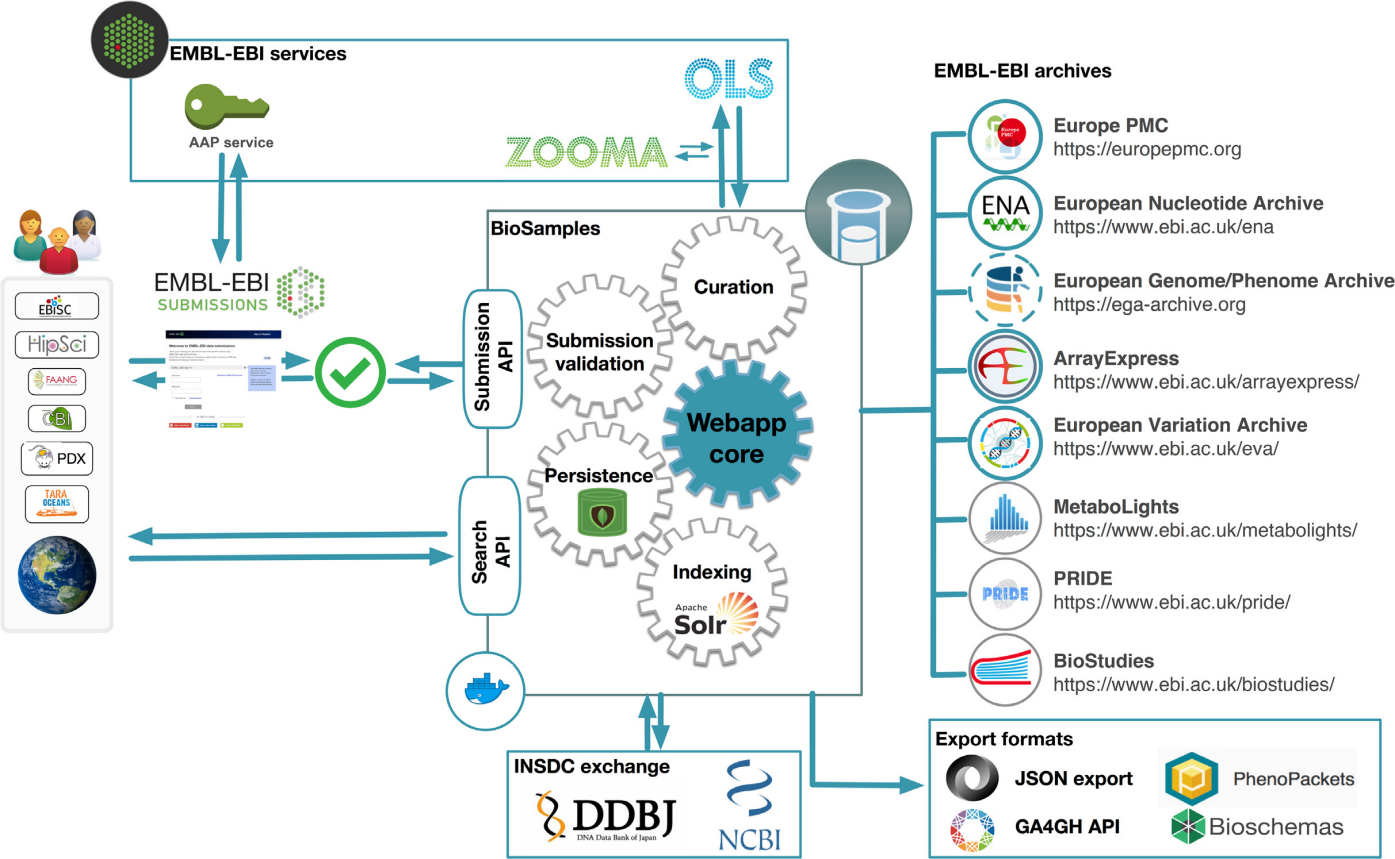
Users can submit and search the BioSamples database via APIs. The BioSamples services perform required operations including data persistence using a MongoDB, indexing via Apache Solr, and curation using external EMBL-EBI services OLS and Zooma. BioSamples are exchanged with INSDC partners and exported in multiple formats. The application also provides links to EMBl-EBI archives (blue circle are established links, grey circles are under development).

For large international consortia it is important that sample metadata is rapidly available to the community. This enables effective data coordination and ensures the most accurate metadata recording for projects where data collection, data sequencing and data analysis are performed at different institutions. It also benefits the community by preventing unnecessary replication of sampling, reproducibility of experiments and fosters collaboration between research groups that can combine research data on complementary sampling into combined functional analyses. By promoting early submission of sample metadata to the BioSamples database, the FAANG project ensures that samples meet its strict metadata requirements, and also prevents data and knowledge loss in the subsequent years of analysis and paper preparation (15). The BioSamples record can then be referenced at the point of later data submission, ensuring accuracy and linkage across different data types. Providing faster turnaround for sample submission has driven major improvements to the BioSamples database infrastructure as shown on Figure 3. To accomplish this, along with increased scalability and resilience, we have upgraded our backend storage, APIs and user interface. A more flexible solution for sample metadata storage in its native JSON format allows the BioSamples database to benefit from the resiliency of cross-datacenter replication, and existing relational database at the core of the BioSamples platform were replaced with a MongoDB™ document database. Rapid availability of samples required an onverhaul of our indexing and retrievals mechanisms. We completely redesigned the interfaces for programmatic access to the BioSamples database, releasing an entirely new RESTful API to supplement the previous downloads of sample metadata. Full documentation for the API is available at https://www.ebi.ac.uk/biosamples/docs/references/api/overview. This new API is available for programmatic users to query and submit metadata to the BioSamples database, and is used to power the web interface and coordinate interaction between components. For example, upon submission of a new sample, an event is raised to notify our metadata indexer, which reads the new sample from our RESTful API and makes it rapidly available for search. This indexer is built upon the Apache Solr™ platform, enabling search queries of large datasets in the BioSamples database. To support improved search both via our UI and APIs, we have leveraged Solr to introduce new filtering and faceting functionality and have redesigned both the website and our search APIs to give users more freedom and flexibility in creating complex queries as shown on Figure 1. This large-scale redesign has allowed us to release an entirely new, more portable version of the software behind the BioSamples database, available as docker image from Quay.io, quay.io/ebibiosamples/biosamples-v4. This makes it possible to easily install a local version of the BioSamples database to host restricted data such as patient-derived omics data in the context of a federated EGA. The entire codebase is open source under the Apache License 2.0, and is available on GitHub, https://github.com/EBIBioSamples/biosamples-v4.

While the new BioSamples database version allows for continuous submission and addresses the need for almost instantaneous availability of samples, subsequent depositions in other archives require the sample metadata to be compliant with established guidelines, such as the ENA guidelines(18). In collaboration with ELIXIR-EXCELERATE and the HCA, we are developing a metadata validator, based on JSON schema, providing extensions to validate ontology annotation. This validator can determine that the value of a metadata attribute matches a term from an ontology, or a subset of that ontology defined by a subclass restriction. For example, it is possible to validate whether a supplied term for the attribute ‘disease’ matches the label of subclass of a the ‘disease’ class ‘EFO:0000408’ from the Experimental Factor Ontology (EFO) (19). This tool is being promoted via an ELIXIR Validation implementation study, which aims at overcome challenges associated with resource-specific validation tools and implementations. The validator will be shared with the HCA Data Coordination Platform and USI projects as an emerging reference implementation for metadata validation, with rules clearly described and implemented in an open service.

Sample metadata, as originally submitted to the BioSamples database, is sometimes insufficiently comprehensive to drive downstream analysis pipelines, for example in the marine metagenomics field (20). By reading the original source papers, metagenomics curators are often able to determine the required missing metadata, but to date have not been able to update BioSamples records to reflect this fact without permission from the original submitter, which is often difficult to obtain. We developed an original system of *curation objects* to enable metadata update (e.g. when curated by collaborators, or when errors are reported by users) without altering the original submission. Curation objects overlay curated information over pre-existing data. Each curation object is dated and owned by a specific AAP domain. This means that the original submission can remain compliant with the checklist it was submitted against, whilst curators and downstream pipelines can take advantage of the additional or enriched metadata. It also ensures that updates and annotations to the sample metadata can be traced and removed if later found inaccurate or inappropriate. The curated sample and the curation objects can be retrieved in both original and curated versions via our API. Curation is supported both via manual or programmatic input as well as through our own automated pipelines to add semantic annotations to all sample metadata. These include annotation using Zooma, https://www.ebi.ac.uk/spot/zooma/, and linking to a wide range of ontology terms via the Ontology Lookup Service (OLS), https://www.ebi.ac.uk/ols/index. Once sample metadata has been annotated with ontology terms, it is enriched for search by expansion to parent ontology classes. This means that attribute values can be leveraged for complex queries, based on the ontology hierarchy. Users can, for example, query for ‘cancer’ and receive all results for subclasses (e.g. ‘lung adenocarcinoma’, ‘leukemia’) as well as synonyms (e.g. ‘lung neoplasm’). When applied to the antibiogram data described above queries can be executed per family of antibiotics, such as penicillin, by taking advantage of automatically added annotation to the Chemical Entities of Biological Interest (ChEBI) ontology(21).

## GLOBAL METADATA INTEROPERABILITY

As genomic data evolve from a research perspective towards the clinical realm and the prospect of personalized medicine gets nearer, the BioSamples database is preparing to better represent phenotypic information, especially as applied to human clinical data. To achieve this, we have established and deployed resources within GA4GH, an international organization devising and leading implementation of new standards to enable genomic data sharing under a responsible framework. In this context we work closely with EGA, a driver project for the GA4GH. The BioSamples database has contributed to the development of the GA4GH data model and an API specification for data export and querying. All human disease related metadata and links to genomics archives are now available through an implementation of this GA4GH API specification. We have collaborated with the Phenopackets developers, http://phenopackets.org, to extend their existing schema to include additional elements required for samples representation, such as geographical location and specimen collection information. A first export of samples using the new Phenopacket format was released in August 2018 and is available from each sample page. We anticipate the format will evolve and be augmented as we identify further required attributes based on the GA4GH use cases, including metadata exchange with the EGA.

A key challenge in bridging clinical and bioinformatics research is closing the gap between biobanks and public data archives. This would allow to provide traceability of samples, including the data that is generated from them, without compromising the anonymity of the patients who donated them. In a typical workflow, samples are physically obtained and stored by biobanks. Researchers can obtain patient samples from biobanks in order to conduct a variety of assays, before submitting their results to a public archive, which may be controlled access depending on the type of assay, as a prerequisite of publication. This data is then made available back to other researchers to subsequently exploit for further analysis. BioSamples accessions can provide this traceability, but to date a mechanism to enable biobanks to acquire BioSamples accessions and easily trace links to datasets has not existed. We now use Bioschemas, an extension to schema.org, that provides a mechanism to expose life science data in a structured form through the web without the need for a dedicated API. We have led the development of the samples specification for BioSchemas and are now exposing a Bioschemas compliant JSON-LD file for each of our samples, https://www.ebi.ac.uk/biosamples/samples/SAMEA229810.ldjson in addition to a general *DataCatalog* description of the repository itself. By bridging between resources (biobanks, curated repositories such as the EGA and the BioSamples database), we enable the possibility of samples being traced back to their source, their combination behind firewalls with privately held genomic data, and the integration of results of other analyses on those same samples in a way that takes into account the need for controlled-access. BioSchemas also provides a mechanism to deliver lightweight metadata exchange pipelines. We earlier discussed the importance of high quality metadata in the metagenomics data. Marine microbial Reference genome database (MarRef) (22) is a highly manually curated resource which ensures metagenomics samples have over 100 metadata attributes accurately described, many more than in a typical BioSamples database submission. In collaboration with MarRef, we are using BioSchemas to mark up additional curated metadata in a manner that the BioSamples database can automatically read, without the need for MarRef to implement a dedicated API. The BioSamples database is now able to incorporate this additional metadata into the original records using the curation object approach we have described above.

## DISCUSSION

To answer our partner’s requirements, the BioSamples database had to dramatically evolve both in terms of data content and technical infrastructure. It has now reached maturity and provides a scalable, resilient, extensible and performant platform over which internal EMBL-EBI teams as well as external groups can now build on to drive better data integration and retrieval from public archives. There is a need to support the increasing number of controlled access datasets, but there are privacy concerns. The containerization of the BioSamples database allows rapid deployment to provide indexing of non identifiable information from controlled access repositories such as EGA. We have engaged with efforts to improve support for querying over controlled-access datasets. Firstly, we are helping to devise methods to automatically tag datasets with information about their consent codes and data use restrictions. We have developed the Data Use Ontology (DUO), http://purl.obolibrary.org/obo/duo.owl, which is already being used by both the Broad institute and the EGA to codify permissions on datasets as well as during data deposition and request at the Sanger Institute. Secondly, we are testing import of the dbGaP metadata from our NCBI BioSample mirror and are planning to add links towards the EGA samples. As a first step, we have provided a pool of accessions to EGA and those have been used to consistently identify their incoming submissions. Our mid term aim is to ensure all appropriately consented human controlled access data has anonymised sample identifiers in the BioSamples database, can be searched using our powerful search tools and ontology integrations, and used to retrieve data from controlled access resource if the appropriate permissions are obtained. The BioSamples database does not currently restrict the types of attributes that can be used to describe sample metadata. Those are therefore often freely chosen by submitters. Whilst this can sometimes hamper comparisons between samples because of differences in the way submitters choose attribute names, it has proven highly effective at getting a large number of submissions with very expressive attributes. We now have ∼45 million sample attributes we can leverage for big data analysis. We have an under-development prototype leveraging text-mining and machine learning methods to semi-automatically improve the information content or our datasets. These will not only help us correct typos and harmonize attributes names (e.g. ‘diseaseStage’, ‘host clinical status’ etc could be labelled as ‘disease’) but also provide a recommendation engine for future submitters. For example, if a submitter enters values for the field latitude/longitude, a recommendation system may choose to advise that other users describing metagenomic data also entered a value for ‘depth’. In conjunction with the validator described above, near matches for existing checklists could also be extracted. This system could advise the same submitter that they were missing the ‘depth’ attribute, and that adding this would make their sample compliant with the TARA ocean checklist, https://www.ebi.ac.uk/ena/submit/tara-oceans-checklist. We have developed a first version of a user interface which allows for human review of proposed curations, and the algorithms learns from the choice curators make. Using this internal prototype, a curator was able to clean 9 million attributes in 20 min for a simple typo identification and correction.We will test this with users and curators of ENA archives and deploy to production as it addresses annotation at scale, a known challenge for biocurators. We would like to push the development of this curation interface into production and use the resulting curated dataset to drive the development of a sample metadata recommendation system, which we plan to make available via an API to services including USI.
